# Comparison Between McCoy Laryngoscope and C-MAC Video Laryngoscope in Anticipated Difficult Airway: A Prospective Randomised Study

**DOI:** 10.7759/cureus.26685

**Published:** 2022-07-09

**Authors:** Mukul Garg, Raju Shakya, Nari Mary Lyngdoh, Debasis Pradhan

**Affiliations:** 1 Anaesthesiology and Critical Care, Dr. Ram Manohar Lohia Hospital, New Delhi, IND; 2 Critical Care Medicine, Indraprastha Apollo Hospitals, New Delhi, IND; 3 Anaesthesiology and Critical Care, North Eastern Indira Gandhi Regional Institute of Health and Medical Sciences (NEIGRIHMS), Shillong, IND; 4 Anaesthesia and Critical Care, University Hospital of Derby and Burton, Derbyshire, GBR

**Keywords:** intratracheal intubation, mccoy, c-mac, laryngoscope, airway

## Abstract

Objective: Prolonged laryngoscopy and failure to intubate are associated with increased morbidity and mortality. Need to improve glottic visualisation and ease of intubation has led to the introduction of various types of laryngoscopes. This study compares the effectiveness of C-MAC video laryngoscope (VL) with McCoy laryngoscope in patients with an anticipated difficult airway.

Methods: This prospective randomised single-blinded single-centre study included patients with modified Mallampati grades 3 and 4, divided into two groups I and II of 65 patients each. Group I was intubated using C-MAC and group II with McCoy Laryngoscope. Modified Cormack Lehane grade of visualisation, time to intubate, intubation difficulty scale score and complications were recorded.

Results: C-MAC VL provides a higher proportion of modified Cormack Lehane grade I visualisation (63% vs 35.3, p=0.0017), the lesser median time of intubation in seconds (15 vs 18, p=0.0007) and significantly lesser median intubation difficulty score (0 vs 3) when compared to McCoy.

Conclusions: C-MAC VL provided better visualisation of glottis and easier tracheal intubation that too in a significantly lesser time. We conclude and recommend the use of C-MAC VL over McCoy for endotracheal intubation in patients with predicted difficult airways, especially in modified Mallampati grades 3 and 4.

## Introduction

Securing the airway by tracheal intubation is a very essential life-saving skill. Complications during tracheal intubation include hypoxia, airway trauma, oesophageal intubation and even cardiorespiratory arrest [1]. Proper alignment of the oro-pharyngeal-laryngeal axis is a key component of successful endotracheal intubation [2]. Failure to intubate is associated with high morbidity and mortality. Surgical airway skills and competencies can be challenging for anesthesia providers which necessitate an improvement in airway management techniques [3,4].

The most popular laryngoscope in the world was developed by Sir Robert Macintosh in 1943. The Macintosh blade is the most commonly used laryngoscope blade, which is curved. Since then, different types of laryngoscopes have been devised to decrease the incidence of failed intubations [5,6]. McCoy laryngoscope is a Macintosh blade with a lever-controlled hinged tip that provides relatively better glottic visualization [7,8]. C-MAC (Karl Storz, Tuttlingen, Germany) is a portable VL that includes an original Macintosh blade, CMOS digital camera at the tip with a light source and a stand-alone video display monitor. The conventional Macintosh blade provides direct and the video component provides indirect visualisation of glottis [9,10]. Although both C-MAC VL and McCoy laryngoscope have been shown to provide better glottic visualisation, there are very few studies that have compared the two laryngoscopes with regard to ease of intubation. C-MAC video laryngoscope (VL) resulted in easier tracheal intubation in patients posted for cervical spine surgery in one study [11] while another study found McCoy laryngoscope to take lesser time for intubation [12]. Therefore we designed a prospective randomised study comparing C-MAC with McCoy in patients with a predicted difficult airway.

## Materials and methods

A prospective randomised single centre study was performed following approval from the institute's ethical committee. The inclusion criteria were the age of 18 to 60 years, receiving general anaesthesia and endotracheal intubation for elective surgery, American Society of Anesthesiologists (ASA) grades I and II, body mass index (BMI) 18.5 to 24.9 and modified Mallampati grades (MMG) 3 and 4. Those patients with pregnancy, BMI > 24.9, inadequate mouth opening (<4 cm), thyromental distance <6 cm, restricted neck movement or any diseases involving the neck, upper respiratory tract and upper alimentary tract were excluded from the study. The following selection and written informed consent taken during preoperative assessment, we randomised patients into two groups based on the sealed envelope technique. Group I was intubated using C-MAC VL and group II with McCoy laryngoscope. Laryngoscopy and intubations were performed by an anesthesiologist who was familiar and trained with intubation using McCoy and C-MAC VL. Patient randomization was stopped when we had data of 65 patients for each group available for final analysis.

We followed the institutional practice guidelines for preop fasting and pre-medications. After explaining the procedure to the patient, standard monitoring (pulse oximeter, electrocardiograph, non-invasive blood pressure and capnography) and good intravenous access were confirmed. The patient's head was kept at the level of the xiphisternum of the anaesthesiologist responsible for airway management.

Preoxygenation was done with 100% oxygen for at least three minutes. Induction was done using intravenous Fentanyl (2 mcg/kg) and Propofol (2 mg/kg or more) followed by the neuromuscular blockade, achieved by intravenous succinylcholine 2 mg/kg. Resolution of fasciculations or end of 90 seconds after succinylcholine injection was considered as the time to do laryngoscopy. Intubation was done in the “sniffing morning air” position, that is extension at the atlanto-occipital joint and flexion of the neck at the lower part of the cervical spine.

Successful intubation was confirmed by bilateral equal air entry, misting of the tube, equal chest rise and continuous quantitative capnography. Intubation difficulty scale score (IDS) was the primary outcome, which included modified Cormack and Lehane (MCL) grading, number of attempts, number of operators, number of alternative techniques, need of lifting force, external laryngeal pressure and mobility of vocal cords. Time for intubation was the time between insertion of the blade into the mouth until detection of end-tidal carbon dioxide on the monitor. 

In case of failure to intubate after two attempts and or any change in the physiological parameters such as bradycardia (<60 per minute) or oxygen saturation < 90%, during induction and intubation, then the patient was dropped out of the study and was managed according to the standard protocol. Complications such as sore throat, desaturation, dental injuries, mucosal injuries and oesophageal intubation, bronchospasm were also noted and treated accordingly. 

Statistical analysis

Based on earlier findings, a change in mean IDS score of 1 with a standard deviation (SD) of 2.00 between two groups was considered clinically important [10]. Considering alpha error to be 0.05 and 80% power, the sample size in each group was estimated to be 63. Data were prepared using Microsoft word and excel (Microsoft Corporation, USA). Statistical analysis was done by using the MedCalc software version (MedCalc Software Ltd., Belgium). Demographic variables were statistically analysed using an unpaired t-test. Kolmogorov-Smirnov test was used to check the normality of data. Continuous data were presented as mean ± SD, ordinal data as median with an interquartile range (IQR) and categorical data as frequency and proportions. Further analyses were performed by using a two-sided unpaired Student’s t-test for continuous data and Fisher’s exact test for categorical data. Statistical significance was considered when p < 0.05.

## Results

Following exclusion as explained under methods, data of 130 patients were available for final analysis. There was no significant difference between the two groups with respect to the baseline characteristics such as age, gender, actual body weight, ASA status (I and II) and MMG ( 3 and 4) (Table 1). Intubation parameters obtained from laryngoscopy such as IDS score, total time taken for intubation, MCL grades are enumerated in Table 2.

**Table 1:** Baseline characteristics of patients of CMAC and McCoy group Values are means (± SD)*, number (percentage), ASA - American Society of Anaesthesiologist, MMG - Modified Mallamapati grade

Parameters	Group I (C MAC) (n=65)	Group II (Mc COY) (n=65)	P-value
Age (year)	38.9 (±10)*	40.9 (±11)*	0.2831
Weight (kg)	54.7 (±10.2)*	56.6 (±5.16)*	0.1768
Sex (male: female), n (%)	(26:39) (40:60)	(33:32) (50.7:49.2)	0.2222
ASA status			
I, n (%)	42 (64.5)	37 (57)	0.5613
II, n (%)	23 (35.5)	28 (43)	0.5148
MMG			
3, n (%)	50 (76.9)	47 (72.3)	0.5484
4, n (%)	15 (23.1)	18 (27.7)	

**Table 2:** Endpoints of laryngoscopy in CMAC and McCoy group Values are in median (IQR) or number (%), IDS - Intubation difficulty scale, MCL - Modified Cormack-Lehane

Parameters	Group I (C MAC) (n=65)	Group II (Mc COY) (n=65)	P value
IDS	0 (0-2.25)	3 (1-4.25)	<0.0001
Intubation time (s)	15 (13-17)	18 (14-20)	0.0007
MCL Grading			
1	41 (63)	23 (35.3)	0.0017
2A	13 (20)	15 (23)	0.6784
2B	8 (12.3)	18 (27.9)	0.0271
3	3 (4.61)	9 (13.8)	0.0710
4	0	0	0
Bronchospasm	2 (3.07)	3 (4.61)	0.649

## Discussion

Difficult airway management remains a challenge despite the invention of many novel airway devices, even in the hands of experienced anaesthesiologists. Endotracheal intubation has two basic steps, exposure of the glottic opening and passage of the tip of the endotracheal tube between the vocal cords. During laryngoscopy, optimum head extension and neck flexion are paramount for adequate alignment of the oro-pharyngeal-laryngeal axis which ascertains visualisation of the glottic opening.

The distal levering tip of the McCoy blade allows an elevation of the larynx from the area of the teeth towards the vallecula leading to better alignment of axes and glottic visualisation [13]. Various VLs have been designed to offer a better view of the glottis with or without proper alignment of the oro-pharyngeal-laryngeal axis. The availability of a more curved D blade makes VLs more useful in glottic visualisation in difficult airway situations [14-18]. Initial evaluations of the C-MAC VL have shown its superiority over the Macintosh laryngoscope [5]. However, there is a paucity of literature on the comparison between C-MAC VL and McCoy laryngoscope. In the present study, baseline characteristics including MMG were similar. MCL grade was noted to assess glottic visualisation along with the time of intubation in both groups. It was observed that visualisation of glottis and ease of intubation was superior with C-MAC than with McCoy. C-MAC VL provided a significantly higher proportion of MCL Grade I visualisations compared to McCoy. This observation is similar to the earlier findings [19]. It was found that the median time to intubation was significantly less in C-MAC compared to McCoy laryngoscope. A thorough search of all the studies was made but very few studies were found comparing ease of intubation and glottis visualisation during laryngoscopy and intubation with C-MAC VL and McCoy laryngoscope [20]. A previous study showed a significantly lesser time for intubation in the McCoy laryngoscopy group as compared to the C-MAC group. This difference can be attributed to the difference in patient population and inter-user variability.

There have been multiple studies comparing standard Macintosh blades, McCoy blades and various VLs. One of the studies, in which they compared tracheal intubation with Macintosh, McCoy and TruViewTM in patients with the immobilised cervical spine, reported better glottic visualisation, a higher rate of successful intubation during the first attempt and easier tracheal intubation, and with TruView as compared to Macintosh and McCoy laryngoscopes [21]. This is similar to the results in the present study even though a different type of VL was used and the study population included was different. This suggests that VLs are better alternatives to direct laryngoscopes in ensuring ease of intubation and better glottic visualisation. AirtraqTM VL was found to be superior when compared with McCoy and Macintosh laryngoscopes, with respect to the ease of intubation and glottic visualisation [22].

A meta-analysis published in 2011 compared VLs with direct laryngoscopes in patients scheduled for elective procedures. It showed that the VL exhibited a significantly shorter duration of endotracheal intubation in patients with difficult airways [23]. Similarly, in the present study, the median intubation time was 15 seconds (IQR: 13-17) in the C-MAC group and 18 seconds (IQR:14-20) in the McCoy group, implying that lesser time was taken for intubation using C-MAC as compared to McCoy with significant difference between two groups. Multiple studies have reported that better glottic visualisation with VL did not always result in easier intubation in all patients [24]. In the present study, patients in the C-MAC group have significantly less median IDS of 0 (IQR: 0-2.25) compared to 3 (IQR: 1-4.25) in the McCoy group with a p-value <0.05, which is similar to previous findings [10,25]. Hence the use of the C-MAC VL in the setting of an anticipated difficult airway with MMG 3 and 4 should lead to a higher rate of successful intubation when compared with McCoy laryngoscopy. The above evidence suggests the effectiveness of C-MAC VL in difficult airway management in routine anaesthesia care.

There are many limitations of the present study. First, the intubating anaesthesiologist could not be blinded to the type of device used, thus the observer's bias could not be completely eliminated. However, the observer recording the data for the time taken to intubate, number of attempts and complications was blinded from the device type and MMP grading. Observations such as MCL grading and lifting force applied are subjective in nature and not known to the observer. Second, the results of the study may vary with the level of experience of the anaesthetist. Third, the hemodynamic parameters during intubation could not be recorded, which could have been a great supplement to the laryngoscopy outcome. Third, the use of style to alter the shape of the endotracheal tube was not taken into account.

## Conclusions

C-MAC VL was found to improve the view of the glottis, take lesser time for intubation and improve IDS score in comparison to the McCoy laryngoscope. Therefore, this study concludes and recommends the use of C-MAC VL in the airway management of patients with predicted difficult airways, especially with MMG 3 and 4. Further studies taking into account the hemodynamic parameters during intubation, the use of stylet and external laryngeal manipulation and the user learning curves will strengthen the evidence for the use of video laryngoscope in all elective and emergency intubations including the anticipated difficult ones.
